# THE ROLE OF A MULTIDISCIPLINARY TEAM IN THE REHABILITATION OF A PATIENT WITH QUADRUPLE AMPUTATION

**DOI:** 10.2340/jrm.v58.44843

**Published:** 2026-01-14

**Authors:** Oleh BURII, Mariia KOSOVSKA, Volodymyr LYKHACH, Nataliia SOROKA, Serhii DUSHENKO, Denys NAHORNYI, Roman OLIINYK

**Affiliations:** Superhumans War Trauma Center, Lviv, Ukraine

Quadruple amputation is a rare outcome of severe trauma or systemic conditions (1, 2), its incidence is rising in Ukraine due to war-related injuries (3, 4). Effective rehabilitation requires early prosthetic fitting and coordinated multidisciplinary care (5–8), yet in Ukraine these services are often separated. The Superhumans War Trauma Center (est. 2023) integrates prosthetic and rehabilitation services within individualized, goal-directed programmes. This case report presents a patient with quadruple amputation and illustrates the impact of team-based rehabilitation on long-term independence (5).

## NARRATIVE

### Timeline

- March 5, 2024: Blast injury, surgical care and rehabilitation in military hospitals.
- January 31, 2025: Admission to Superhumans War Trauma Center.
- April 25, 2025: Discharge.

### Patient information

A 55-year-old male serviceman sustained injuries in a mortar explosion in combat. He lived with his wife, who provided primary support.

### Amputation levels *(Fig. 1)*

- Right transfemoral amputation.
- Left transtibial amputation.
- Bilateral transradial amputations.

**Fig. 1:**
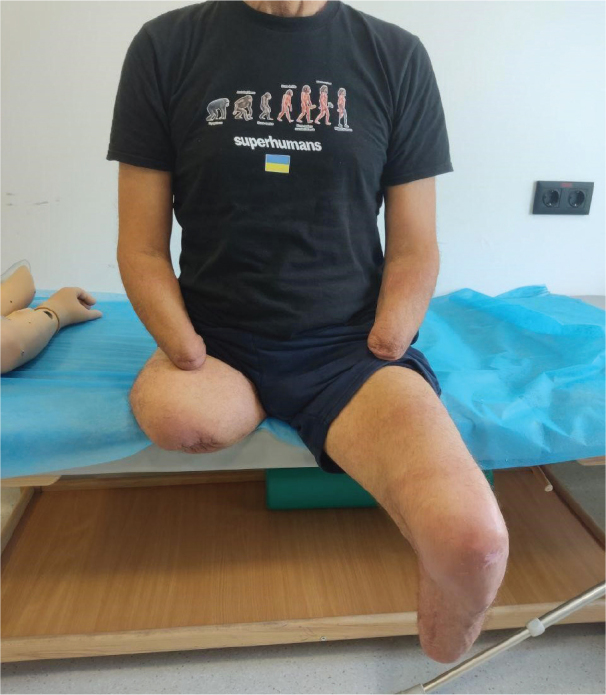
Patient with quadruple amputations.

### Associated conditions

The patient sustained a penetrating TBI of the left frontal lobe with subsequent decompression and cranial reconstruction using a titanium plate (11 May 2024).

### Diagnostic assessment

*PM&R physician*. On admission, the Physical Medicine and Rehabilitation (PM&R) physician diagnosed the patient with residual limb hypersensitivity, cognitive impairment due to frontal-lobe TBI, bilateral sensorineural hearing loss, and functional impairments. The patient was already using lower and upper limb prostheses from another facility.

*Physical therapist*. On admission, the patient exhibited significant limitations in functional mobility (Tables I and II).

**Table I:** Functional assessment

Assessment tool	Score
AMP-B	25 pts, K1 level
Houghton Scale	3/12
PLUS-M (12-item)	29/60
LCI-5	26/56

**Table II:** Functional tests (performed using a single forearm crutch)

Test	Result
TUG	32 s
4SST	21 s
6MWT	195 m
10MWT	(0.8 m/s)
Stair Climb Test (10 steps)	Ascent 36 s, descent 55 s

TUG: Timed Up and Go test. 4SST: Four Square Step Test. 6MWT: Six-Minute Walk Test. 10MWT: Ten-Minute Walk Test.

Gait analysis using Kinovea revealed the following deviations (Video 1):

- *Sagittal plane:* step length asymmetry (right > left), reduced right knee flexion (~15°) during swing, prolonged first rocker at initial contact, and upper limb asymmetry due to crutch use.
- *Frontal plane:* moderate trunk lean to the right, right leg circumduction during swing, and external foot rotation at contact.

*Occupational therapist.* At baseline, the patient was dependent in hygiene and partially dependent in feeding, and unable to functionally use upper limb prostheses; Rancho Level IX indicated purposeful activity with limited self-management.

*Psychologist.* PHQ-9, GAD-7, and PCL-5 showed no clinical depression, anxiety, or PTSD, though mild avoidance symptoms were present. A MoCA score of 17 out of 30 indicated moderate cognitive impairment, with reduced attention and slowed processing. The patient’s wife showed signs of caregiver burden.

*Vocational counsellor.* An internally displaced person with quadruple limb loss, the patient could not return to his previous work; together with his wife, he explored vocational reintegration options.

## THERAPEUTIC INTERVENTION

### PM&R physician

PM&R led the individualized SMART-based rehabilitation programme, ensuring coordinated, goal-directed holistic care (5). Treatment included cardiopulmonary load monitoring under sinus bradycardia, treatment of right olecranon bursitis, dental care for apical periodontitis, hearing-adapted communication, presbyopia correction, and cognitive-load regulation after frontal-lobe injury.

### Physical therapy

The patient completed intensive rehabilitation (54 sessions, 5 sessions/week, 2 h/session), with a focus on gait, balance and endurance training, and functional mobility tasks, alongside 12 hydrotherapy sessions.

Early goals focused on fall-risk reduction and safe ambulation with one crutch (6MWT ≥ 250 m, gait speed ≥ 1.0 m/s). His prosthetics were upgraded to better fit functional needs. Training progressed to stair and ramp negotiation, outdoor walking on uneven terrain, and reduced assistive device use. Final goals targeted community ambulation (6MWT ≥ 450 m, gait speed ≥ 1.6 m/s) and prosthesis use > 8 h/day.

### Prosthetics: lower limbs

**Fig. 2:**
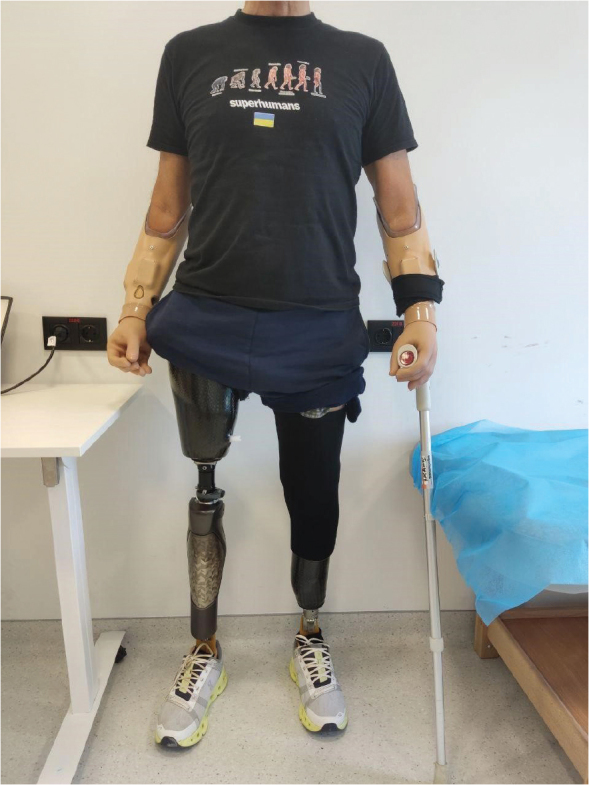
Prosthetic components, lower limbs.

The patient completed staged fitting for definitive prostheses (10 sessions). Components were upgraded to a C-Leg 4 MPK (Ottobock, Duderstadt, Germany) and Maverick feet (Ottobock) with ICS and TSB sockets with a vacuum system and an external sleeve on the TT side (Fig. 2).

### Prosthetics: upper limbs

**Fig. 3:**
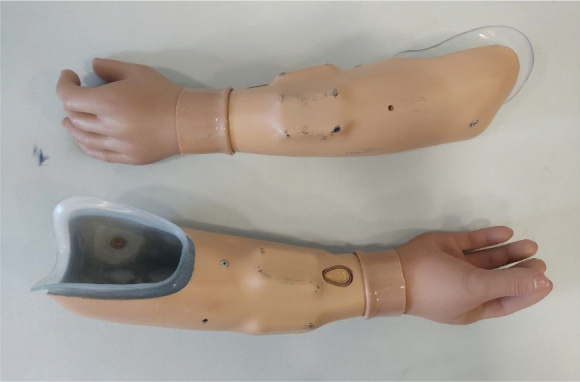
Prosthetic components, upper limb.

The patient underwent staged prosthetic fitting (12 sessions), receiving 2 myoelectric forearm prostheses (Fig. 3):

- Inner socket: Northwestern-style.
- Outer socket: 3D-printed made from PA12 material.
- Hand prosthetics: MyoHand Variplus Speed (Ottobock) and Greifer DMC Vari Plus (Ottobock).

### Occupational therapy

Over 11.6 weeks, the patient received 58 occupational therapy sessions. Training focused on the independent donning and doffing of upper and lower limb prostheses, eating, hygiene, and simple manual tasks.

### Psychologist

The psychologist coordinated interventions with physical therapy and provided support to reduce anxiety, address body image concerns, and maintain motivation for prosthesis use. Neurocognitive stimulation continued for TBI-related deficits, and the wife received psychoeducation to prevent caregiver burnout.

### Vocational counselling

The patient received 2 consultations focused on vocational readaptation and social reintegration. Options included starting a business, adapted employment, and veteran programmes. His wife was prepared to help manage a potential business.

## FOLLOW-UP AND OUTCOMES

### Physical therapy

The patient progressed to K3 mobility with an AMP-B score of 37, walking independently with a single cane and wearing prostheses 4–8 h/day. Functional mobility improved across all measures (Houghton 6/12, PLUS-M 46/60, LCI-5 42/56), indicating enhanced gait stability, endurance, and independence (Table III).

**Table III:** Functional test results on discharge

Test	Result	Improvement
TUG	15.6 s	-16.4 s
4SST	11 s	-10 s
6MWT	360 m	+165 m
10MWT	(1.4 m/s)	+0.6 m/s
Stair Climb Test (10 steps)	Ascent 16 s, descent 29 s	Ascent –20 s, descent –26 s

For abbreviations, see Table I.

### Gait analysis on discharge

Gait improvements included restored step symmetry, right knee flexion of 50–60°, smooth heel-to-toe rollover, and normalized upper limb movements. In the frontal plane, no gait deviations were observed (Video 2).

### Occupational therapy

On discharge, the patient demonstrated basic manual dexterity, transferring 10 blocks with the right and 8 with the left prosthesis in the Box and Block Test. He could independently don and doff his prostheses, perform hygiene routines, and use them for eating and household tasks.

### Psychological support

Support improved the patient’s emotional stability and engagement in therapy, while strengthening the wife’s coping capacity as primary caregiver. Remaining risks include housing instability, financial strain, and the patient’s emotional dependence.

### Vocational counselling

The patient and his wife received guidance on vocational reintegration, with a veteran-owned business identified as the main option, digital skills training, and information on assistance programmes.

### Patient perspective

“After losing all four limbs, I was in a very difficult state. At Superhumans, I was treated with dignity and received individualized rehabilitation focused on learning to use my prostheses. Now I walk independently, use my bionic arms for daily tasks, and lead an active, fulfilling life again.”

## DISCUSSION

Previous case reports show that early prosthetic fitting, multidisciplinary collaboration, and psychosocial support contribute to favourable outcomes in quadruple amputees (2 ,6, 9). In this case, goal-oriented prosthetic fitting and individualized rehabilitation enabled safe gait restoration and growing independence. Family involvement, especially the spouse as primary caregiver, ensured continuity of care, while vocational counselling supported social reintegration – an aspect seldom noted in earlier reports (6, 7, 10). This case also underscores the leadership role of the PM&R physician in coordinating prosthetic, cognitive, and psychosocial care (5).

In conclusion, this case illustrates that functional recovery is achievable after quadruple limb loss, even with cognitive and sensory impairments. Early rehabilitation, advanced prosthetic fitting, family support, and coordinated multidisciplinary care improved independence and community mobility.

## Supplementary Material




